# 5G-Based Telerobotic Ultrasound System Improves Access to Breast Examination in Rural and Remote Areas: A Prospective and Two-Scenario Study

**DOI:** 10.3390/diagnostics13030362

**Published:** 2023-01-18

**Authors:** Tian He, Yin-Ying Pu, Ya-Qin Zhang, Zhe-Bin Qian, Le-Hang Guo, Li-Ping Sun, Chong-Ke Zhao, Hui-Xiong Xu

**Affiliations:** 1 Department of Medical Ultrasound, Center of Minimally Invasive Treatment for Tumor, Shanghai Tenth People’s Hospital, Ultrasound Research and Education Institute, Clinical Research Center for Interventional Medicine, School of Medicine, Tongji University, Shanghai 200072, China; 2 Shanghai Engineering Research Center of Ultrasound Diagnosis and Treatment, Shanghai 200072, China; 3 Department of Medical Ultrasound, Chongming Second People’s Hospital, Shanghai 202157, China; 4 Department of Ultrasound, Zhongshan Hospital, Fudan University, Shanghai 200032, China

**Keywords:** telerobotic operation, tele-ultrasound, 5G network, breast scanning, rural and remote areas, mobile car

## Abstract

Objective: Ultrasound (US) plays an important role in the diagnosis and management of breast diseases; however, effective breast US screening is lacking in rural and remote areas. To alleviate this issue, we prospectively evaluated the clinical availability of 5G-based telerobotic US technology for breast examinations in rural and remote areas. Methods: Between September 2020 and March 2021, 63 patients underwent conventional and telerobotic US examinations in a rural island (Scenario A), while 20 patients underwent telerobotic US examination in a mobile car located in a remote county (Scenario B) in May 2021. The safety, duration, US image quality, consistency, and acceptability of the 5G-based telerobotic US were assessed. Results: In Scenario A, the average duration of the telerobotic US procedure was longer than that of conventional US (10.3 ± 3.3 min vs. 7.6 ± 3.0 min, *p* = 0.017), but their average imaging scores were similar (4.86 vs. 4.90, *p* = 0.159). Two cases of gynecomastia, one of lactation mastitis, and one of postoperative breast effusion were diagnosed and 32 nodules were detected using the two US methods. There was good interobserver agreement between the US features and BI-RADS categories of the identical nodules (ICC = 0.795–1.000). In Scenario B, breast nodules were detected in 65% of the patients using telerobotic US. Its average duration was 10.1 ± 2.3 min, and the average imaging score was 4.85. Overall, 90.4% of the patients were willing to choose telerobotic US in the future, and tele-sonologists were satisfied with 85.5% of the examinations. Conclusion: The 5G-based telerobotic US system is feasible for providing effective breast examinations in rural and remote areas.

## 1. Introduction

Ultrasound (US) is a unique medical imaging technology that plays an important role in the diagnosis and management of breast diseases, owing to its advantages such as real-time scanning, convenience, and radiation-free features [1]. Breast cancer is not only the most frequently diagnosed cancer but also the leading cause of cancer-related death among women worldwide [2]. Women have a 12.3% risk of developing breast cancer during their lifetime [3]. Notably, men are also at a risk of possibly developing breast cancer, with a prognosis worse than that of women [4]. Compared to advanced breast cancer, early breast cancer is considered potentially curable [5]. Routine breast imaging is important for the detection of early breast cancer. Mammography and US are the most common screening modalities used for breast cancer detection. The density of mammary glands is higher in Asian women than in Western women; hence, US is more suitable for breast examination than mammography in Asian women [6,7,8]. Moreover, US is the primary imaging tool for definitive diagnosis, severity assessment, and treatment effect evaluation of non-tumour breast diseases, such as mastitis and gynecomastia [9,10].

Effective breast US examination is crucial to obtain credible results, particularly in some developing Asian countries with inadequate medical resources. However, US examination is highly operator-dependent, and there is a lack of experienced sonologists in rural and remote areas [11]. Consequently, many patients in these areas have to travel to large cities to access high-level medical care in large hospitals. This could lead to delayed diagnosis and treatment and increased economic burden on patients as well as increased medical resource pressure on higher-level hospitals [12].

The current medical resources are far from sufficient to meet this requirement, especially in rural and remote areas. Telemedicine has been developed to solve the problem of unbalanced distribution of medical resources [13]. Many studies have demonstrated that telemedicine can reduce the need for long-distance transportation of patients, leading to time and cost savings for the patients [14,15]. Tele-US, a branch of telemedicine, transmits US images through wired or wireless networks from remote areas to large hospitals for consultation with experienced sonologists [16]. Additionally, the telerobotic US system allows experienced sonologists in large hospitals to operate the transducer remotely and transmit US images in real-time through satellite, terrestrial, or broadband links [17,18].

Early research on different types of commercial robotic US systems has been published [19,20,21]. A series of studies by Adams et al. demonstrated that it is feasible to use a commercial telerobotic US system (MELODY system with a three degrees of freedom [DOFs] manipulator) for adult abdominal and obstetric examinations [19,20]. However, in the MELODY system, the tele-sonologist cannot control the pressure or movement of the transducer, thus requiring an on-site assistant to hold the robotic arm and apply pressure to the patient’s body. A 2009 study by Boman et al. presented the development and technical assessment of the concept of cardiovascular consultation utilising long-distance, real-time echocardiography with the aid of the Medirob Tele system with a serial robot as a diagnostic tool in rural areas [21]. Nevertheless, this system is specific to echocardiography and not widely used in clinical practice. 

In comparison with previous other telerobotic US systems, a new generation of telerobotic US system (MGIUS-R3) with a greater number of DOFs and fifth generation (5G) data modules enables a tele-sonologist to remotely and in real-time control the subtle movements of the US transducer (rotating, rocking, and tilting) in the scanning area by manipulating the dummy US probe. Meanwhile, advances and popularisation in 5G mobile communication technology have further promoted the development of telerobotic US for clinical applications. During the coronavirus disease (COVID-19) outbreak and pandemic, the use of the same type of 5G-based telerobotic US system for cardiopulmonary assessment of patients in the isolated ward or intensive care unit was reported in several studies [22,23,24]. 

However, the usefulness of 5G-based telerobotic US in breast examinations has not been elucidated. The implementation of this technology may provide a unique solution for breast examinations in rural and remote areas with limited medical resources. Thus, we designed a prospective and controlled study to assess the clinical value of the 5G-based telerobotic US system in breast examination in two different scenarios, a rural island and a remote county, to expand the scope of its application.

## 2. Materials and Methods

### 2.1. Study Design

This prospective study adhered to the tenets of the Declaration of Helsinki, and it was approved by the Institutional Review Board of Shanghai Tenth People’s Hospital (NO. SHSY-IEC-4.1/21-334/01). Informed consent was obtained from all the enrolled participants. This prospective research study is registered at www.chictr.org.cn (ChiCTR2100043936) accessed on 5 March 2021.

### 2.2. Study Cohort

Patients from Chongming Island, who were referred to Chongming Second People’s Hospital for breast US examination from September 2020 to March 2021, were consecutively recruited for Scenario A. Chongming Second People’s Hospital on Chongming Island, China is located 72 km away from Shanghai Tenth People’s Hospital, a tertiary referral centre in the central zone of Shanghai, China. The inclusion criteria were age ≥18 years and ≤80 years and agreement to participate in the study with signed informed consent provided. The exclusion criteria were robotic arm failure of the telerobotic US system and incomplete US imaging data. Finally, 63 patients who underwent both conventional and 5G-based telerobotic breast US examinations were enrolled in this study for Scenario A. 

With the same inclusion and exclusion criteria applied for Scenario A, 20 patients from Anji County who underwent 5G-based telerobotic breast US examinations were recruited in May 2021 for Scenario B. Anji County, Zhejiang Province, China is located 220 km away from Shanghai Tenth People’s Hospital. 

### 2.3. Sonologists and On-Site Assistant

Six sonologists and one on-site assistant participated in this study. Five tele-sonologists with 5–20 years of work experience in breast US scanning conducted 5G-based telerobotic US examinations at Shanghai Tenth People’s Hospital in central Shanghai. An on-site sonologist with 15 years of work experience in breast US scanning performed conventional US examinations at Chongming Second People’s Hospital, Chongming Island. The on-site assistant, a hospital auxiliary personnel with one year of work experience, was in charge of guiding the patient examination in an orderly manner, operating the telerobotic US patient-side subsystem, applying coupling agents, protecting the patient’s privacy, and recording the examination time.

Before the study, each tele-sonologist attended a theoretical learning session on operating the telerobotic US doctor-side subsystem. Meanwhile, the on-site assistant attended a theoretical learning session on operating the telerobotic US patient-side subsystem and the basic anatomy of the breast and axilla. With the help of the on-site assistant, each tele-sonologist independently and completely executed telerobotic breast US scanning for three additional volunteers by following a standardised breast US examination protocol.

### 2.4. 5G-Based Telerobotic US System

**Figure 1:**
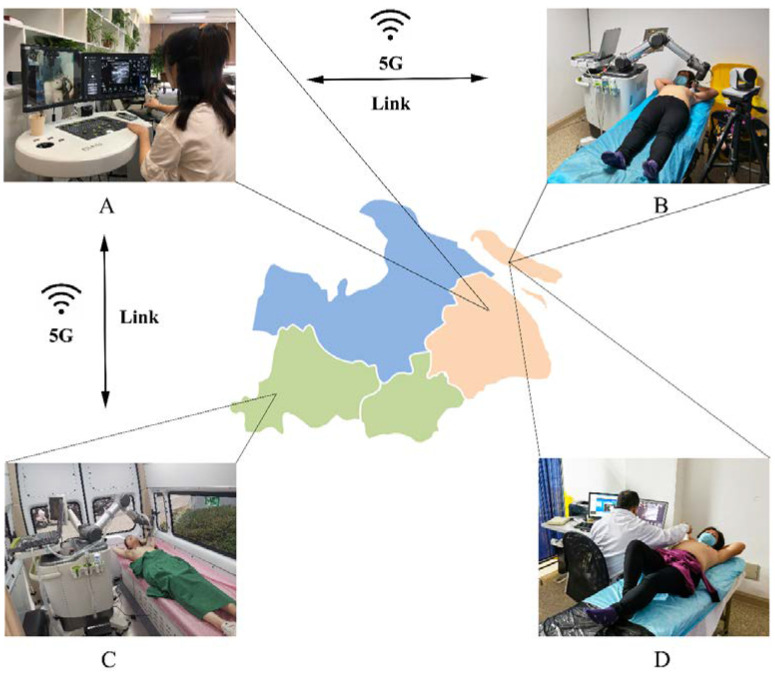
Illustration of the doctor-side subsystem and patient-side subsystem of the 5G-based telerobotic ultrasound system in two different scenarios. (**A**) The tele-sonologist at Shanghai Tenth People’s Hospital manipulates a dummy US probe to perform 5G-based telerobotic breast US examinations remotely for (**B**) the patients in Chongming Second People’s Hospital of Chongming Island and (**C**) the patients in a mobile car located in Anji County. (**D**) An on-site sonologist performs conventional breast US examination for the patients in Chongming Second People’s Hospital. (US, ultrasound; 5G, fifth generation).

This study adopted a commercial telerobotic US system (MGIUS-R3; MGI Tech Co., Ltd., Shenzhen, China), which included a doctor-side subsystem and a patient-side subsystem (Figure 1). This telerobotic US system was a complete set of equipment that obtained European CE certification, Australian Drug Administration certification, and China CFDA certification. The two subsystems were connected by a 5G network (China Mobile Communications Corporation, Shanghai, China). The telerobotic US system included a commercial QueCtel 5G data module. The transmission between the 5G data modules and the host computer occurred through the M.2 Key-B interface (PCI-E 3.0 2X, up to 1.97 Gbps and approximately 15 Gbps, including the USB 2.0/3.0 interface for dialling and receiving AT commands for dialling). On the other hand, mobile data communication was applied between the 5G data module and 5G base station. The 5G communication had multi-frequency band coverage at the same time, while the 5G data module had a corresponding frequency management mode that could conduct frequency searches from high to low frequencies. The microbase stations operated in a frequency band of approximately 5 GHz. In this frequency band, the maximum download and upload speeds were 930 Mbps and 130 Mbps, respectively. The end-to-end delay of the remote parameter tuning was less than 200 ms. The latest fabrication materials and technologies of radiofrequency devices were used to address the potential issue of the high frequency used in 5G, such as conversion efficiency, linearity, and directionality in the process of converting electrical energy to electromagnetic waves.

The doctor-side subsystem of Shanghai Tenth People’s Hospital in central Shanghai was equipped with a robot control console, US imaging control system, and audio–video communication system (Figure 1). The robot control console consisted of a mobile dummy US probe (built-in gesture sensor and “UP” button) and a contact plate (built-in position and pressure sensors). By associating the robot coordinate system with the robot control console coordinate system and motion transformations, the robot control console could manage six DOFs of the robotic arm to achieve the desired movement of the probe. Thus, the action of the operator was consistent with the action of the robotic arm. The gesture sensor managed three DOFs for rotation, the position sensor managed two DOFs for the movement on the horizontal plane, and the pressure sensor and “UP” button managed one DOF for the down and up movements, respectively. Using the US system control panel on the doctor’s side, all parameters and functions of the US imaging system, including gain, depth, focus, measurement, and colour Doppler parameters on the patient’s side subsystem, could be adjusted and implemented in real-time by the tele-sonologist. During the telerobotic US examination, the quantified transmission delay was measured and dynamically displayed on the screen to the remote doctor. The colour of the icon was green when the delay time was less than 100 ms, yellow when it was 100–500 ms, and red when it was greater than 500 ms. It could assist the tele-sonologist in determining the delay status.

The same patient-side subsystems were located in Chongming Second People’s Hospital of Chongming Island and in a mobile car parked in Anji County (Figure 1). The patient-side subsystem was equipped with a six-DOFs collaborative robotic arm (UR5; Universal Robots, Odense, Denmark), a portable US imaging system with a 5–12 MHz linear array transducer (Wisonic Clover 60; Huasheng Medical Systems, Shenzhen, China), and an audio–video communication system. The 6-DOF robotic arm could conduct six-dimensional motion in space to control the posture of the US transducer. The positioning accuracy was up to 0.1 mm. A force sensor at the front-end robotic arm provided real-time force feedback information. During the interaction between the US transducer and the human body, the force sensor recorded three-dimensional (3D) force information in real time. The vertical component of the 3D force was fed back to the controller as the actual contact force. Additionally, the sensitivity of the contact force could be accurate to 0.1 N. Meanwhile, the magnitude of the contact force was displayed synchronously and dynamically on the screen of the doctor-side subsystem to the tele-sonologist. Screen imaging of the US machine and scene, including the motion of the robotic arm, position of the US transducer, and posture of the patient, shot by an angle-adjustable camera with the function of amplification, could be transmitted to experts in real time and dynamically displayed using the doctor-side subsystem to the tele-sonologist.

This system had multiple protective designs to ensure patient safety. The robotic arm had maximum limit settings for the moving speed (≤0.275 m/s) and contact pressure (5 N), and the patient-side subsystem had an emergency stop function for the robotic arm. If the robotic arm was out of control, the assistant would press the emergency stop button, and the robotic arm would stop immediately due to power stoppage to the robot. Furthermore, the robot motion stopped within 250 ms if the robotic arm detected that the collision force exceeded 120 N.

### 2.5. 5G-Based Telerobotic Breast US Examination Protocol

In Scenario A, the patients first underwent conventional breast US examination by an on-site sonologist at Chongming Second People’s Hospital, using the same US imaging system (Wisonic Clover 60) as the reference standard. Subsequently, telerobotic breast US examinations were performed by the tele-sonologist at the Shanghai Tenth People’s Hospital for these patients. Both types of US examinations followed a standardised breast examination protocol [25]. The tele-sonologists and on-site sonologists were blinded to each other’s US examination findings. In Scenario B, the patients only underwent telerobotic breast US examinations by the tele-sonologist at Shanghai Tenth People’s Hospital. 

The specific examination procedure of the 5G-based telerobotic breast US examination is described here. Before the examination, the on-site assistant attached the US linear array transducer to the robotic arm, and the tele-sonologist checked the 5G network connectivity and control of the robotic arm. The on-site assistant requested a breast US examination, registered the basic information of the patients (including name, sex, and age) in the patient-side system, and entered it in the doctor-side system. The tele-sonologist then received the examination request, adjusted the examination mode to breast scanning, and asked the patients for their chief complaint and medical history via the audio–video communication system. The patients took off their clothes and laid down on the examination bed in the supine position, with their arms raised overhead. 

**Figure 2:**
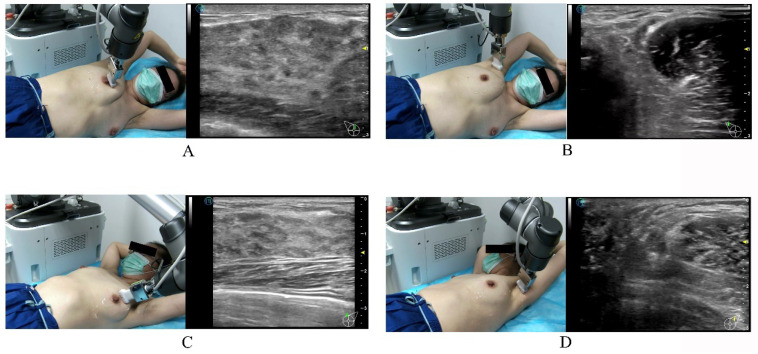
Illustration of the positions of the patient for 5G-based telerobotic breast ultrasound examination. When scanning the right breast (**A**) and axilla (**B**), the patient is placed in the left lateral position with the arms raised overhead. When scanning the left breast (**C**), the patient is placed in the supine position with the arms raised overhead. While scanning the left axilla (**D**), the patient is in the right lateral position with the arms raised overhead.

The tele-sonologist instructed the patient to assume the appropriate breast examination posture with the help of the on-site assistant via the audio–video communication system. The on-site assistant applied adequate coupling agents to the bilateral breasts and axillae of the patient. The tele-sonologist activated the examination button, and the robot arm was moved over the patient. The on-site assistant then dragged the US transducer and positioned it on the patient’s breast (quick positioning). By manipulating the dummy probe, the tele-sonologist then scanned the four quadrants of the breast and the nipple and axilla (Figure 2). 

**Figure 3:**
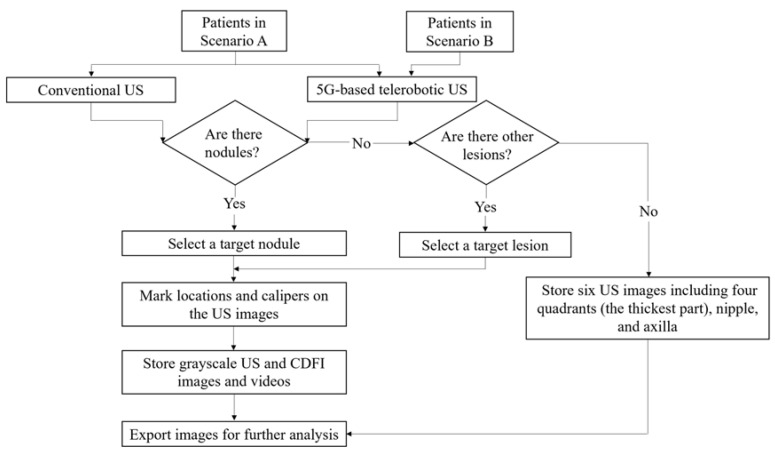
Flowchart of 5G-based telerobotic breast ultrasound examination in the two scenarios.

For both conventional and 5G-based telerobotic US examinations, the information described below was noted in the protocol registration (Figure 3). For each breast, at least six US images including the four quadrants (the thickest part), nipple, and axilla were stored. If one or more breast nodules were detected, then only the largest nodule or the one most suspicious of malignancy on US was chosen as the target nodule. If other breast lesions were detected, the most clinically significant lesion was selected as the target lesion. At least three US images (one grayscale US image, one grayscale US image with callipers, and one colour Doppler flow image) and a video of the target nodule/lesion at the largest cross-section were stored. When storing the US image, a picture of the overall external environment of the transducer’s position was also stored for further analysis.

The durations of both the conventional and 5G-based telerobotic US procedures were recorded from when the patient was ready on the examination bed to be examined to the end of image collection. Additionally, all data collection and organisation were completed by an independent coordinator. All patients were assigned sequential numbers prior to the examination, and folders containing the US images were named according to those numbers.

### 2.6. US Images Interpretation

Two sonologists with 12 and 13 years of work experience interpreted all the US images randomly. A consensus was reached after discussion in case of disagreement between the two interpreting sonologists. 

The assessment of the US images was performed in two steps. First, the quality of all the US images was scored using a subjective quality scoring method [26]. The scoring was as follows: 1 point: very poor (image quality is severely impaired); 2 points: poor (image quality is impaired); 3 points: fair (image quality hinders viewing slightly but acceptable for interpretation); 4 points: excellent (minor suggestions for improvement but viewing is unhindered); 5 points: perfect (no suggestion for improvement). Second, the target breast nodules were assessed. The US characteristics and categories of the breast nodules were assessed based on the Breast Imaging Reporting and Data System (BI-RADS) of the American College of Radiology [27]. 

### 2.7. Patients and Tele-Sonologists’ Assessments

After each 5G-based telerobotic US examination, the patients and tele-sonologists were asked to complete a corresponding questionnaire about their experience of the 5G-based telerobotic US examination. 

### 2.8. Statistical Analysis

Statistical analysis was performed using Statistical Product and Service Solutions (version 20.0; IBM Corporation, Armonk, NY, USA). The measurements of the same breast nodules and axillary lymph nodes were compared between the conventional and telerobotic US findings using a paired-sample t-test. The intraclass correlation coefficient (ICC) with confidence intervals was calculated to evaluate the consistency between the US features and BI-RADS categories of the same breast nodules and interpreted as follows: ICC > 0.90 indicated excellent consistency, ICC = 0.75–0.90 indicated good consistency, ICC = 0.50–0.74 indicated moderate consistency, and ICC < 0.50 indicated poor consistency. Two-tailed *p*-values < 0.05 indicated a statistically significant difference.

## 3. Results

### 3.1. Patient Demographics and Examination Information

The mean age of the patients in this study (*n* = 83; 2 males and 81 females) was 50.7 ± 13.1 years (range, 24–72 years). Among them, 11 females and 1 male belonged to the young-aged group of 20–34 years, 28 females to the middle-aged group of 35–49 years, and 44 females and 1 male to the old-aged group of 50–80 years. 

For Scenario A, 63 patients (61 females and 2 males; average age, 53.5 ± 13.0 years) were enrolled in this study. Of these, 36 (57.2%) patients underwent US scanning for health checks, 13 (20.6%) for follow-up examination for breast masses, 12 (19.0%) for breast pain, 1 (1.6%) for follow-up of postoperative plasma cell mastitis, and 1 (1.6%) for acute mastitis.

For Scenario B, 20 patients (20 females; average age, 41.8 ± 8.7 years) were enrolled. Of these patients, 16 (80%) patients underwent US scanning for health check and 4 (20%) for the follow-up examination for breast masses (Table 1).

### 3.2. Safety and Duration of 5G-Based Telerobotic Breast US Examinations

During the telerobotic breast US examination, none of the participants had any injuries in either scenario, highlighting the safety of the 5G-based telerobotic US system.

The average duration for the 5G-based telerobotic breast US examinations was 10.3 ± 2.7 min (range, 5–22 min). In Scenario A, the average durations for the 5G-based telerobotic US examinations and conventional US examinations were 10.3 ± 3.3 min (range, 5–22 min) and 7.6 ± 3.0 min (range, 4–16 min), respectively. Overall, the average duration of the 5G-based telerobotic US examination was approximately 2.7 min longer than that of the conventional US examination. In Scenario B, the average duration for the 5G-based telerobotic US examinations was 10.1 ± 2.3 min (range, 8–14 min).

### 3.3. 5G-Based Telerobotic Breast US Findings

**Figure 4:**
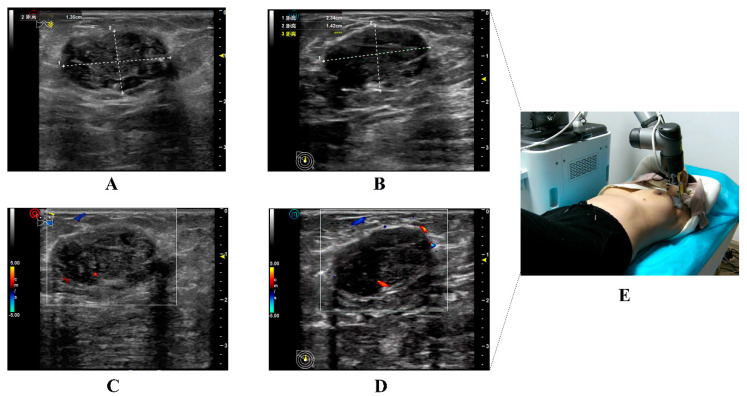
Illustration of the ultrasound images of the same target nodule in a 36-year-old woman obtained from conventional and 5G-based telerobotic ultrasound examinations. Grayscale US images of the same breast nodule from the (**A**) conventional US examination and (**B**) 5G-based telerobotic US examination. The US characteristics of the breast nodule are as follows: oval shape, parallel orientation, circumscribed margins, hypoechoic echo pattern, and posterior shadowing with no calcifications. The nodule is classified as BI-RADS 4. Colour Doppler flow images are from the (**C**) conventional US examination and (**D**) 5G-based telerobotic US examination. There are blood flow signals detected inside this breast nodule. (**E**) The transducer is located in the outer upper quadrant of the left breast. (US, ultrasound; 5G, fifth generation; BI-RADS, Breast Imaging Reporting and Data System).

In Scenario A, 34 breast nodules were detected using 5G-based telerobotic US and 35 using conventional US. Moreover, 32 breast nodules identified on 5G-based telerobotic US examination were consistent with those detected on conventional US examination (Figure 4). In addition to the breast nodules, two cases of gynecomastia, one of lactation mastitis, and one of postoperative breast effusion were diagnosed using both these US procedures.

The 5G-based telerobotic US examinations missed three breast nodules classified as BI-RADS 3. Among them, one breast nodule was located in the outer quadrant of the left breast in a 72-year-old woman, and two breast nodules were located in the outer quadrant of the right breast in two obese women with body mass indexes of 33 and 34. Conventional US examinations missed two breast nodules classified as BI-RADS 3 (Table 2).

In Scenario B, breast nodules were detected in 65% of the patients (13 of 20) using 5G-based telerobotic US. There were 11 breast nodules (mean transverse diameter of 7.6 mm and anteroposterior diameter of 3.8 mm) classified as BI-RADS 3, and 2 breast nodules (mean transverse diameter of 6.1 mm and anteroposterior diameter of 5.3 mm) classified as BI-RADS 4. Two patients with suspicious malignant breast nodules belonged to the middle-aged and old-aged groups, respectively. 

### 3.4. US Images Quality Assessment of 5G-Based Telerobotic Breast Examinations

Two sonologists scored all the US images using a five-point Likert scale. The average US image quality score of the 5G-based telerobotic US was 4.86. In Scenario A, the average US image quality scores of the 5G-based telerobotic and conventional US systems were 4.86 and 4.90, respectively. A paired-sample t-test found no significant difference between them (*p* = 0.159) (Table 3). Moreover, 88.9% of the cases assessed using 5G-based telerobotic US had a score of 5 points. In Scenario B, the average US image quality score of the 5G-based telerobotic system was 4.85. This result indicates that the 5G-based telerobotic US system can obtain high-quality US images.

### 3.5. Consistency between the Findings of 5G-Based Telerobotic and Conventional US Examinations in Scenario A

A paired-sample *t*-test revealed no significant differences between the 5G-based telerobotic and conventional US examinations in the transverse and anteroposterior diameter measurements of the same breast nodules and axillary lymph nodes (Table 4). Good inter-observer agreement was observed in the US features of the same breast nodules for the parameters of shape, orientation, margin, echo pattern, posterior features, calcifications, and BI-RADS category between the 5G-based telerobotic and conventional US examinations (ICC = 0.893, 0.795, 0.874, 1.000, 0.963, 0.882, and 0.984, respectively) (Table 5). 

### 3.6. Patients’ Assessments

In total, 91.6% of patients enrolled in the two scenarios reported no discomfort or uneasiness during the 5G-based telerobotic US examination, and 94% of the patients were not afraid of the robotic arm. Only one female had obvious tenderness in the bilateral whole breast. Furthermore, 92.7% of females considered the duration of the 5G-based telerobotic US examination as strongly or somewhat acceptable. Of the patients, 90.4% were willing to undergo 5G-based telerobotic US examination, and 89.2% were willing to pay an extra fee for it in the future. Overall, the 5G-based telerobotic US system was well accepted by the patients (Table 6).

### 3.7. Tele-Sonologists’ Assessments

The tele-sonologists reported no obvious delay or difficulty during the majority of 5G-based telerobotic US examinations (97.6% and 81.9%, respectively). However, they expressed concern in the scope of scanning of patients with large breasts. The tele-sonologists were satisfied with the duration of 86.7% of telerobotic US examinations, and 85.5% of US images were transmitted from the 5G-based telerobotic US system. Of the tele-sonologists, 84.3% were willing to use the 5G-based telerobotic US system as a routine US examination tool (Table 6).

## 4. Discussion

Our work possesses several strengths. To the best of our knowledge, this is the first prospective and two-scenario study exploring the practical value of the 5G-based telerobotic US system in breast scanning and diagnosis. We propose a standardised breast US examination protocol for the 5G-based telerobotic US system that can efficiently check the bilateral whole breast and axillae. It will facilitate standardised breast US examinations as well as training and clinical development of this cutting-edge technology. Through the application of 5G network technology, 83 breast US examinations were successfully completed with the use of a telerobotic US system diagnostic system in two scenarios at a community hospital located on a rural island and a mobile car located in a remote county, respectively. In terms of the safety, duration, US image quality, consistency with conventional US results, and acceptability for patients and tele-sonologists, this study demonstrates that a 5G-based telerobotic US diagnostic system has a relatively high level of feasibility for the diagnosis and management of breast diseases.

Many patients in rural and remote areas with limited medical resources are forced to go to higher-level hospitals for US examinations. In Scenario A, there were only two large community hospitals on Chongming Island, with a high proportion of patients in the older age groups. Chongming Second People’s Hospital, one of the two hospitals in this study, has only two junior sonologists dedicated for US. In Scenario B, hospitals or imaging centres with limited medical resources were not available in Anji County. Chongming Island and Anji County are 72 km and 220 km away from central Shanghai, respectively, corresponding to at least 1.5 h and 3 h of one-way driving time without traffic jams, respectively. In Scenario A, the average duration of 5G-based telerobotic breast US examinations was approximately 26% longer than that of conventional US examinations (10.3 ± 3.3 min vs. 7.6 ± 3.0 min). This is consistent with the result of a previous study by Arbeille et al., which found that the duration of teleoperated foetal US examinations was approximately 30% longer than that of the conventional US examinations [28]. Notably, the average duration of the 5G-based telerobotic breast US examinations in Scenario A (10.1 ± 2.3 min) was similar to that in Scenario B (10.3 ± 3.3 min). Despite the slightly increased examination time with 5G-based telerobotic US, most patients found it acceptable, as reported in the questionnaire survey conducted in our study. Considering the need for the corollary equipment and an on-site assistant, the examination cost of the 5G-based telerobotic US system is higher than that of the conventional US system. However, from a societal perspective, 5G-based telerobotic US examination as a distant diagnosis strategy will result in an overall cost reduction owing to a reduction in the travel-related expenses of the patients [29]. Moreover, the questionnaire survey revealed that most patients from the two scenarios had a high acceptance of the 5G-based telerobotic US system and were willing to pay an additional examination fee. Thus, 5G-based telerobotic US will help save travel time and expenses for patients from rural and remote areas lacking medical resources.

In addition, 5G-based telerobotic US can also effectively minimise the transport of large numbers of patients to higher-level hospitals and relieve the burden on these hospitals. In light of the global spread of COVID-19 and the emergence of new viral variations, it is important to reduce unnecessary human transportation to prevent and control the epidemic. Furthermore, sonologist groups can provide subspecialty expertise to a greater number of underserved communities to expand their reach and create additional revenue opportunities. Additionally, work-related musculoskeletal disorders are prevalent among sonologists, with previous studies indicating that approximately 90% of sonologists complain of musculoskeletal discomfort or pain when conducting US scans [30]. By reducing the scanning forces applied by sonologists and equipping an ergonomic arm cushion, a 5G-based telerobotic US system could help relieve occupational chronic musculoskeletal injuries. 

During 5G-based telerobotic breast US examination, none of the patients suffered any injuries, and 98.8% reported no discomfort in both scenarios. This system has dual protection devices to ensure patient safety and provides real-time force feedback information from the force sensor visualised as a column to ensure complete patient comfort. Nevertheless, one female patient (1.2%) experienced obvious breast tenderness during telerobotic US examination, probably because she was premenstrual, which made the bilateral whole breast regions swollen and sensitive [31]. Thus, inquiries regarding the patient’s menstrual cycle should be routinely made, and the contact force applied should be appropriately reduced in those who are menstruating or premenstrual.

Two-way and real-time data communication of the telerobotic US system between the tele-sonologist and patient sites requires a large broadband capacity. These data flows generally include robot control, US system control, force feedback, US videos, and multiple audio–visual signals. US video data requires more bandwidth than the other data, which affects the performance of the US diagnosis to a certain extent [17,32,33]. Arbeille et al. used a robotic arm to perform remote abdominal US examinations via a bandwidth network of 250 Kbps, and the degradation of US image quality led to six (17%) cases of missed lesions [17]. Our study indicated that the quality of US images transmitted from the telerobotic US system over the 5G network was comparable to that of conventional US system, fulfilling the diagnostic demand. The transmission speed (peak rate of up to 20 Gbps) of the latest 5G network is 1.5 times better than that of the current 4G network, and the delay (approximately 1–10 ms) is reduced by a factor of 10 [34]. The application of 5G telecommunication technology substantially increases the data transmission capacity and resolves the issues of latency, which allows long-distance original US image acquisition, transmission, analysis, and processing, with high-precision synchronisation of multiple audio–visual signals. 

In Scenario A, two cases of gynecomastia, one of lactation mastitis, and one of postoperative breast effusion were diagnosed and 32 breast nodules were detected using the two US methods. Moreover, there were no major differences between the parameters measured using the two US methods. The 5G-based telerobotic US detected 92.7% of the breast abnormalities. This indicates that the 5G-based telerobotic US system has high diagnostic performance. The 6-DOF robotic arm facilitates fine breast examination. The tele-US control panel could be used to achieve remote adjustment of the US parameters, including frequency, gain, depth, and parameter measurement. However, three breast nodules were missed in the 5G-based telerobotic US examination. When storing the US image, a picture of the overall external environment of the transducer position was also stored. By analysing the photographs and US data, the potential reasons for the missed nodules were investigated. Poor contact between the US transducer and a loose skin surface of older female patients might have increased the chances of incomplete breast scanning. A greater amount of coupling agents should be applied to ensure optimum contact force. Moreover, it is difficult to scan large-sized breasts in obese patients. Although the 6-DOF arm can move flexibly along the contours of the human body, it still has restrictions on the side of the body, resulting in difficulties in scanning the outer quadrant of the breast. It is necessary to adjust the patient’s position to achieve appropriate contact with the transducer on the side [22]. This would be beneficial not only for the location of breast lesions but also in follow-up examinations. Both static US images and dynamic videos were stored during the examination, which was conducive for repeated continuous observations. In contrast, two breast nodules were detected only with the 5G-based telerobotic US system and not with the conventional US system. This could be attributed to the operator’s dependency on US examination [20]. 

In Scenario B, the positive disease detection rate in the mobile car group was 65%. Among these females, two with suspicious malignant breast nodules (BI-RADS 4) were recommended to undergo further examinations and treatments. Takeuchi et al. reported the testing of a mobile robotic tele-echo system placed in an ambulance that successfully transmitted clear real-time echo images of the patient’s abdomen to the destination hospital from where this device was being remotely operated through private networks [33]. Owing to its unique advantages, the mobile car equipped with a 5G-based telerobotic US system can be parked in several remote areas where hospitals or imaging centres are not available. This technology holds the potential to allow patients to stay in their home community for US examinations while improving their access to imaging expertise offered at larger centres.

The 5G-based telerobotic US system is still in its prime and requires further advancements. It does not yet provide the realistic functions of haptic feedback and 3D information. Haptic feedback restoration in medical robotics platforms is gaining growing attention. It is also based on the recent integration of haptic feedback in the well-known da Vinci system, which can show how the operator can make better decisions [35]. Thus, additional investigations based on force sensing might also help overcome the occurrence of uncomfortable conditions in patients. Meanwhile, the two-dimensional vision information from the camera obviously affects 3D space perception during breast examination. The degree of immersion often referred to as the “sense of being there” experienced by the operators is a factor in the success of tele-health installations. Further efforts can use high-definition 3D video conferencing technology, which can offer a compelling mechanism to achieve a sense of immersion and contribute to an enhanced quality of use [36,37]. Furthermore, to achieve remote US instrument control, a special US imaging system was selected. We hope this device can be compatible with more brands of US imaging system in the future, which will help generalize this technology.

Despite the positive outcomes of our study, it has several limitations. First, it was a single-center study with limited-size data. Multicenter and large-scale studies are needed to validate our results. Second, there was inherent subjectivity in our simplified questionnaire survey for the tele-sonologists. This was for rapidly evaluating the direct feelings of tele-sonologists during the telerobotic US examinations, but might result in inaccurate assessment. Third, the 5G-based telerobotic US examination was not compared with the conventional US examination in the mobile car setting (Scenario B). However, these differences were highlighted in Scenario A, and the purpose of this study was to consider the clinical practice management considerations for implementing 5G-based telerobotic US in a real-world setting. Finally, the tele-sonologists in the doctor-side subsystem in this study were all senior sonologists. We did not investigate the telerobotic breast US examination results of tele-sonologists with different work experiences, nor did we determine whether experienced tele-sonologists could easily master the telerobotic US system. 

## 5. Conclusions

This research preliminarily demonstrates that a 5G-based telerobotic US system can achieve comparable quality images and diagnostic results for breast examination over the conventional US system. Moreover, it provides a novel and potential solution for the application of quality breast US in rural areas lacking experienced sonologists or in remote areas where hospitals or imaging centres are not available. It might help to provide increased access to breast examination in rural and remote areas and greater equity in the delivery of health care services. Currently, artificial intelligence (deep learning and machine learning methods) studies are rapidly evolving and have many potential applications in breast US, such as lesion detection, BI-RADS classification, breast cancer risk prediction, and breast cancer molecular subtype prediction [38,39,40,41]. Further studies about integrating artificial intelligence technology into the 5G-based telerobotic US system would greatly facilitate the detection and diagnosis of breast disease accurately and efficiently and increase the application scope of this technology. 

## Figures and Tables

**Table 1:** Basic information of enrolled patients. Except where indicated, the data in parentheses are percentages. †: The data are expressed as means ± standard deviation, with ranges in parentheses.

Characteristics	Total	Scenario A	Scenario B
No. of patients	83	63	20
Gender			
Female	81 (97.6)	61 (96.8)	20 (100.0)
Male	2 (2.4)	2 (3.2)	0 (0.0)
Mean age (years) †	50.7 ± 13.1 (24–72)	53.5 ± 13.0 (24–72)	41.8 ± 8.7 (27–55)
20–34 years	11 (13.3)	5 (7.9)	6 (30.0)
35–49 years	28 (33.7)	17 (27.0)	11 (55.0)
50–80 years	44 (53.0)	41 (65.1)	3 (15.0)
Complaint			
Health check	52 (62.6)	36 (57.2)	16 (80.0)
Follow-up for breast masses	17 (20.5)	13 (20.6)	4 (20.0)
Breast pain	12 (14.5)	12 (19.0)	0 (0.0)
Follow-up with plasma cell mastitis	1 (1.2)	1 (1.6)	0 (0.0)
Acute mastitis	1 (1.2)	1 (1.6)	0 (0.0)

**Table 2:** Missing breast nodules in the 5G-based telerobotic and conventional US in Scenario A. BMI: calculated as weight (kg)/height (m)^2^. Abbreviations: US: ultrasound; BI-RADS: Breast Imaging Reporting and Data System. BMI: body mass index.

Characteristics	Missing Nodules in the Telerobotic US			Missing Nodules in the Conventional US	
	Nodule 1	Nodule 2	Nodule 3	Nodule 1	Nodule 2
Age (years)	50	42	72	45	59
BMI	33	34	25	25	28
Clinical sign	No sign	Breast tenderness	No sign	Breast tenderness	No sign
Side (right/left)	R	R	L	L	R
Size of breast (cup)	C	D	B	B	C
Transverse diameter (mm)	5	6	15	5	5
Anteroposterior diameter (mm)	4	3	5	4	3
BI-RADS category	3	3	3	3	3

**Table 3:** Quality scoring of 5G-based telerobotic US images and conventional US images. Except were indicated, data in parentheses are percentages. †: The data are expressed as means ± standard deviation, with ranges in parentheses. Abbreviations: US: ultrasound.

Parameter		Total	Scenario A			Scenario B
		Telerobotic US (*n* = 83)	Conventional US (*n* = 63)	Telerobotic US (*n* = 63)	*p* Value	Telerobotic US (*n* = 20)
Average examination time (minutes) †		10.3 ± 2.7 (5–22)	7.6 ± 3.0 (4–16)	10.3 ± 3.3 (5–22)	0.017	10.1 ± 2.3 (8–14)
Image quality scoring (5-point Likert scale)	Average score	4.86	4.90	4.86	0.159	4.85
	1 point	0 (0.0)	0 (0.0)	0 (0.0)		0 (0.0)
	2 points	0 (0.0)	0 (0.0)	0 (0.0)		0 (0.0)
	3 points	3 (3.6)	0 (0.0)	2 (3.2)		1 (5.0)
	4 points	6 (7.2)	6 (9.5)	5 (7.9)		1 (5.0)
	5 points	74 (89.2)	57 (90.5)	56 (88.9)		18 (90.0)

**Table 4:** Measurements of the same breast nodules and axillary lymph nodes detected by 5G-based telerobotic and conventional US examinations in Scenario A. † Data are means ± standard deviation, with ranges in parentheses. Abbreviation: US, Ultrasound.

Measurement	Telerobotic US	Conventional US	*p* Value
Same breast nodules (*n* = 32)			
Transverse diameter (mm) †	10.3 ± 7.8 (4.0–39.2)	9.9 ± 7.3 (3.0–37.0)	0.140
Anteroposterior diameter (mm) †	6.4 ± 5.7 (2.5–23.2)	4.9 ± 3.4 (2.5–19.0)	0.061
Same axillary lymph nodes (*n* = 16)			
Transverse diameter (mm) †	16.3 ± 6.4 (9.0–30.8)	15.2 ± 6.9 (8.0–27.4)	0.296
Anteroposterior diameter (mm) †	8.1 ± 4.1 (3.6–21.5)	7.2 ± 1.9 (4.0–21.5)	0.344

**Table 5:** Interobserver agreement in US features of the same breast nodules in Scenario A. Except were indicated, data in parentheses are percentages. *: Numbers in parentheses are 95% confidence intervals. Abbreviations: US: ultrasound; ICC: intraclass correlation coefficient.

US Characteristics	Telerobotic US	Conventional US	ICC *	
Shape			0.893 (0.781, 0.948)	
Oval/round	27 (84.4)	25 (78.1)		
Irregular	5 (15.6)	7 (21.9)		
Orientation			0.795 (0.579, 0.900)	
Parallel	30 (93.8)	31 (96.9)		
Not parallel	2 (6.2)	1 (3.1)		
Margin			0.874 (0.742, 0.938)	
Circumscribed	28 (87.5)	27 (84.4)		
Not circumscribed	4 (12.5)	5 (15.6)		
Echo pattern			1.000 (1.000, 1.000)	
Anechoic	5 (15.6)	5 (15.6)		
Hypoechoic	24 (75.0)	24 (75.0)		
Complex cystic and solid	3 (9.4)	3 (9.4)		
Posterior features			0.963 (0.924, 0.982)	
Shadowing	15 (46.9)	15 (46.9)		
Enhancement	7 (21.9)	8 (25.0)		
No posterior features	10 (31.2)	9 (28.1)		
Calcifications			0.882 (0.759, 0.942)	
No calcifications	29 (90.6)	30 (93.8)		
Calcifications	3 (9.4)	2 (6.2)		
BI-RADS category			0.984 (0.967, 0.992)	
2	5 (15.6)	5 (15.6)		
3	20 (62.5)	19 (59.4)		
4	6 (18.8)	7 (21.9)		
5	1 (3.1)	1 (3.1)		

**Table 6:** Answers of patients and tele-sonologists to the questionnaires in the two Scenarios. Except where indicated, data in parentheses are percentages.

	Strongly Agree	Somewhat Agree	Neutral	Disagree
Patients				
(1) I do not feel discomfort during the 5G-based telerobotic US examination	76 (91.6)	6 (7.2)	0 (0.0)	1 (1.2)
(2) I am not afraid of the robotic arm during the 5G-based telerobotic US examination	78 (94.0)	0 (0.0)	3 (3.6)	2 (2.4)
(3) I am satisfied with duration of the 5G-based telerobotic US examination	49 (59.0)	28 (33.7)	0 (0.0)	6 (7.3)
(4) I accept to be examined by 5G-based telerobotic US system in future	75 (90.4)	0 (0.0)	4 (4.8)	4 (4.8)
(5) I am willing to pay an extra fee for the 5G-based telerobotic US examination in future	74 (89.2)	4 (4.8)	0 (0.0)	5 (6.0)
Tele-sonologists				
(1) I feel no transmission delay during the 5G-based telerobotic US examination, which does not affect the US scanning.	81 (97.6)	2 (2.4)	0 (0.0)	0 (0.0)
(2) I have no difficulty in operating the 5G-based telerobotic US system	68 (82.0)	9 (10.8)	6 (7.2)	0 (0.0)
(3) I am satisfied with the transmitted US images from the 5G-based telerobotic US system	71 (85.5)	7 (8.4)	5 (6.1)	0 (0.0)
(4) I don’t think it would take long to perform the 5G-based telerobotic US examination in comparison with estimated conventional US examination	72 (86.7)	10 (12.1)	1 (1.2)	0 (0.0)
(5) I am willing to use the 5G-based telerobotic US system as routine US examination tool	70 (84.3)	11 (13.3)	0 (0.0)	2 (2.4)

## Data Availability

The datasets generated and analysed during the current study are not publicly available but are available from the corresponding author on reasonable request.
